# Brass Alloys: Copper-Bottomed Solutions against Hospital-Acquired Infections?

**DOI:** 10.3390/antibiotics10030286

**Published:** 2021-03-10

**Authors:** Emilie Dauvergne, Catherine Mullié

**Affiliations:** 1 Laboratoire AGIR-UR UPJV 4294, UFR de Pharmacie, Université de Picardie Jules Verne, 80037 Amiens, France; emilie.dauvergne7@laposte.net; 2 FAVI Limited Company, 80490 Hallencourt, France; 3 Laboratoire Hygiène, Risque Biologique et Environnement, Centre Hospitalier Universitaire Amiens-Picardie, 80025 Amiens, France

**Keywords:** copper, brass, antibacterial activity, hospital acquired infections, antibiotic resistance, antibacterial surfaces

## Abstract

Copper has been used for its antimicrobial properties since Antiquity. Nowadays, touch surfaces made of copper-based alloys such as brasses are used in healthcare settings in an attempt to reduce the bioburden and limit environmental transmission of nosocomial pathogens. After a brief history of brass uses, the various mechanisms that are thought to be at the basis of brass antimicrobial action will be described. Evidence shows that direct contact with the surface as well as cupric and cuprous ions arising from brass surfaces are instrumental in the antimicrobial effectiveness. These copper ions can lead to oxidative stress, membrane alterations, protein malfunctions, and/or DNA damages. Laboratory studies back up a broad spectrum of activity of brass surfaces on bacteria with the possible exception of bacteria in their sporulated form. Various parameters influencing the antimicrobial activity such as relative humidity, temperature, wet/dry inoculation or wear have been identified, making it mandatory to standardize antibacterial testing. Field trials using brass and copper surfaces consistently report reductions in the bacterial bioburden but, evidence is still sparse as to a significant impact on hospital acquired infections. Further work is also needed to assess the long-term effects of chemical/physical wear on their antimicrobial effectiveness.

## 1. Introduction

Brasses are alloys composed of copper and zinc, as opposed to bronze which are alloys of copper and tin. The zinc percentage within brass alloys can vary from 5 to 40% [1]. Below 35% of Zn, the alloy is termed copper-rich and forms a single solution of face-centered cubic (fcc) copper matrix called α brass [1,2]. Above 35% of Zn, an ordered body-centered-cubic (bcc) phase called β phase is stably formed in addition to the α phase [1,2]. From a layman point of view, copper-rich brass alloys are called red brasses, while brasses with a higher Zn content are termed yellow brasses [3]. Over time, other chemical elements such as manganese, nickel, aluminum, lead or silicium, to name a few, have been added to brass to create the High Entropy Alloys (HEA) used nowadays, improving their technological properties and resistance to corrosion [2,4].

Along with bronze, brass has been one of the first alloys forged by mankind with reports of brass artifacts dating back to the 3rd millennium BC [5]. In Europe, the first intentional productions of brass in the Greek, Etruscan and Roman civilizations are thought to have taken place sometime during the 1st millennium BC [6]. Brass was then used to create everyday objects such as handles, coins, arms (daggers, axes, etc.) or decorative objects such as fibulae, rings and statuettes [6]. In addition to these early mundane uses of brass alloys, and more in line with the subject of this paper, copper, the main component of brass, has also long been employed to prevent water fouling or treat infections, with a first written trace of such uses found in the Smith papyrus (around 1500 BC) [7]. Practices such as the storage of Ganges’ water in brass or copper utensils in accordance with ancient texts of Ayurveda in India or the addition of copper coins in water canteens by Second World War Japanese soldiers to keep the water sanitary have been documented, indirectly pointing at the bactericidal activity of copper [8,9,10]. This bactericidal activity is concentration-dependent. Indeed, at low concentrations, copper plays an important role in the life of bacteria as it is involved in a number of their metalloenzymes called cuproenzymes [11,12]. However, at high concentrations, copper ions overcome the bacterial handling/detoxification systems and are able to generate a wide array of detrimental perturbations for the bacterial cell such as mismetalation of metalloenzymes induced by the ionic imbalance or excessive production of reactive oxygen species (ROS) initiated through a Fenton-like reaction. Those ROS can in turn lead to membrane, protein and DNA damages and eventually to cell death [12,13].

In healthcare settings, the initial observations on the potential of brass to limit the bacterial contamination of touch surfaces, as compared to stainless steel, were published in the early eighties [14]. These observations paved the way towards a possible mean of reducing indirect transmission of bacteria originating from touch surfaces, especially in healthcare facilities. It thus offered an additional preventive tool in the fight against hospital acquired infections (HAIs).

The mechanisms underlying the bactericidal effect of touch surfaces made of copper and copper alloys have since been explored. A number of laboratory and field studies have been performed to better characterize the effectiveness of copper and its alloys on nosocomial pathogens and on the prevention of HAIs. The aim of this review is to summarize the current knowledge in these areas, with a focus on brass alloys.

## 2. Antimicrobial Mechanism of Action of Copper-Containing Surfaces

Observational descriptions of the bactericidal effect of surfaces made out of copper or copper alloys were reviewed as early as in the seventies [15]. Many laboratory studies on various bacterial species followed, showing a rapid (within minutes) bactericidal effect of copper-containing surfaces against most of the strains tested.

**Figure 1:**
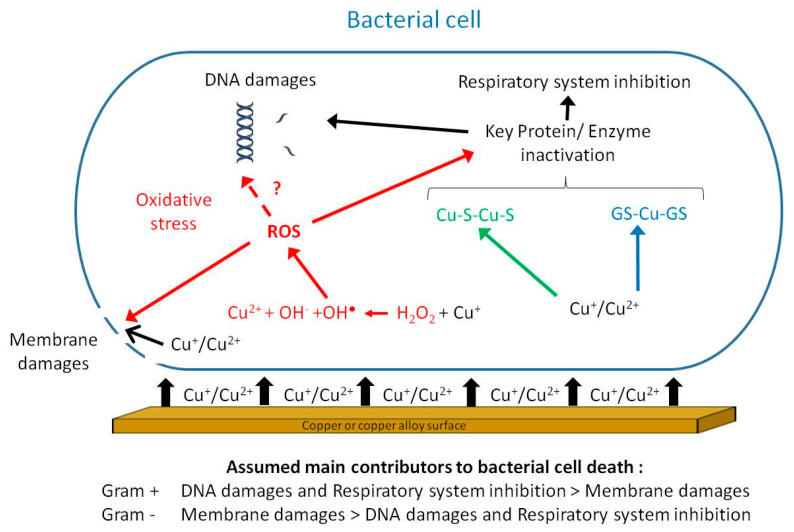
Putative mechanisms involved in contact killing: direct destruction of the membrane by cupric and cuprous ions (black pathway); hydrogen peroxide-dependent oxidation of Cu^+^ in the cell under aerobic conditions generating reactive oxygen species (ROS) (red pathway); interactions between copper ions and glutathione under anaerobic conditions (blue pathway) and displacement of iron from iron-sulfur clusters (green pathway). The final steps leading to bacterial cell death include inactivation of key proteins/enzymes, among which are those involved in the respiratory system as well as membrane and DNA damages.

This biocide activity has been termed contact killing [16]. Contact killing implies a direct and mandatory contact between bacteria and the surface for the antibacterial activity to take place. This point was demonstrated by the lack of antibacterial activity of copper coupons coated with a honeycomb-like polymer grid preventing direct contact with a wet *Enterococcus hirae* inoculum [17]. Meanwhile, similar but uncoated copper coupons were able to induce a reduction in the *E. hirae* inoculum greater than 10^6^ bacteria in 30 min. Importantly, copper ion release was found to be similar for both the coated and uncoated coupons. Also, Zheng et al. [18] reported that CuSO_4_ in solution was less effective in killing *Candida albicans* planktonic or biofilm cells than copper ions released from the surface of coupons, implying that direct contact was instrumental in the killing process [18]. A few years later, Solioz proposed a 4-step chronological order for contact killing by copper-containing surfaces [19]. The first and crucial step consists in the dissolution of copper ions from the surface and their accumulation in the small aqueous space between the material surface and bacterial membrane, reaching the mM range [19,20]. These copper ions can lead to (i) the generation of reactive oxygen species (ROS) [21], (ii) the inhibition of the respiratory chain [22], (iii) lipid peroxidation [23,24] damages to cell membrane [24,25,26,27], (iv) DNA degradation [28,29], (v) modified protein expression [30], and (vi) displacement of iron-sulfur clusters and inactivation of metalloproteins [31,32] (Figure 1). The actual mechanism(s) as well as the precise order in which these mechanisms are involved in copper-mediated contact killing is expected to be dependent on factors such as the bacterial physiology and environmental conditions (e.g., presence of moisture or buffering/proteinaceous agents). Nevertheless, copper ions are instrumental in the contact-killing process, as demonstrated by the notable reductions/delays in bacterial deaths registered when copper chelators such as bichinchoninic acid, ethylenediaminetetraacetic acid (for Cu^2+^) or bathocuproine disulfonic acid (for Cu^+^) are used [21,32,33].

### 2.1. Generation of ROS by Copper Ions through a Fenton-Like Reaction

A Fenton-like reaction (1) is often cited as the initial step leading to the biocide effect of copper through the production of the highly reactive hydroxyl radical OH^•^ [16,19,21,25,34].

Cu^+^ + H_2_O_2_ → Cu^2+^ + OH^−^ +OH (1)

Cuprous (Cu^+^) and cupric (Cu^2+^) ions implied in this reaction come from the metal surface [35]. Under the biosphere conditions, a majority of copper-containing contact surfaces release Cu^2+^ when in contact with microorganisms. This release is thought to be enhanced by the surface oxidation/corrosion under ambient conditions (e.g., moderate humidity and air) [35,36]. Regarding H_2_O_2_, the presence of which is mandatory for the completion of Equation (1), it is a natural byproduct of aerobic growth in prokaryotes [37]. Another intracellular source of H_2_O_2_ is the reduction of superoxide radicals (O_2_^•−^) by superoxide dismutases (SOD) [37]. Additionally, according to the Hard–Soft Acid Base (HSAB) theory, Cu^+^ and Cu^2+^ can lead to thiol and gluthatione depletion [38]. Another possibility for H_2_O_2_ production is therefore reactions leading to the depletion of sulfhydryl groups (R-SH) through a cycle between Equations (2) and (3) [16,39]_._

2 Cu^2+^ + 2 RSH → 2 Cu^+^ + RSSR + 2H^+^ (2)

2 Cu^+^ + 2 H^+^ + O_2_ → 2 Cu^2+^ + H_2_O_2_ (3)

HSAB-induced glutathione depletion can in turn leave proteins and other molecules within the microbial cell vulnerable to oxidative stress. It can even prevent the repair of oxidized proteins by cellular thiol–disulphide exchange enzymes. Whatever the origin of H_2_O_2_ in the cell, copper ion-derived production of ROS results in increased oxidative stress. Its consequences on the microbial cell can be multiple and its typical targets are membrane, proteins, and DNA. The actual in vivo formation of hydroxyl radical has been evidenced in *Escherichia coli* [40]. Furthermore, experiments using substances known to interfere with oxidative stress (e.g., catalase, superoxide dismutase or OH^•^ quencher mannitol) were shown to delay contact killing in *E. coli* [21]. However, under anaerobic conditions, bacterial cell death on copper-containing surfaces through contact killing occurs as fast as, if not faster than, in aerobic conditions [33]. Therefore, some authors put forward the hypothesis that cellular ROS-induced damages cannot be the main mechanism behind lethal cellular alterations leading to bacterial death and have proposed alternative mechanisms, such as membrane and/or DNA damages or direct protein function impairment [19,39].

### 2.2. Membrane Damages

The contact-killing process is quite fast and has been shown to occur as early as within 30 s of exposure to copper surfaces [41]. As cell wall/membrane is the bacterial part directly in contact with the surface, it is a likely target when one is looking to a rapid antimicrobial mechanism of action. Indeed, membrane damages have been described for a wide range of microorganisms in contact with copper-containing surfaces [25,26,27,42]. However, a few reports failed to register copper surface-induced membrane alterations [43].

ROS-dependent and independent mechanisms have been proposed to explain these damages. A first possibility is the formation of copper/lipid complexes via binding of copper ions by the hydrophilic heads of phosphatidylethanolamine (PE) and phosphatidylserine (PS) present in the bacterial membrane [37]. During *Bacillus subtilis* cell division, PE domains have been shown to concentrate at the septal region [44]. The complexion of PE with copper ions could interfere with this process and alter the replication process [37]. The binding of cuprous and/or cupric ions to lipopolysaccharide, peptidoglycan and/or carboxylic groups in the bacterial membrane can also induce membrane depolarization [45]. Membrane depolarization is indeed thought to be the main mechanism of bactericidal activity by some authors [25,27]. This phenomenon leads to a leakage of cytoplasmic content and ultimately to the complete rupture of the membrane and cell death.

Polyunsaturated fatty acids are a well-known target of oxidative stress through a radical chain reaction initiated by OH. However, the bacterial cell wall mostly contains saturated and monounsaturated fatty acids, which are less prone to oxidative stress than polyunsaturated ones. Nevertheless, in *E. coli*, contact killing has been linked with an increase in lipid peroxidation witnessed by Thiobarbituric Acid Reactive Susbtances measurement peaking after a 30 min exposure to copper and brass alloys [23]. Also, an *E. coli* mutant strain harboring higher amounts of unsaturated fatty acids was found to be more susceptible to contact killing with copper-containing alloys than its native counterpart [23].

### 2.3. Protein Damages

A proteomic analysis of proteins impacted by dry exposure to copper surfaces revealed that the expression of 210 proteins was modulated [29]. Additionally, a higher degree of oxidative proline and threonine modifications were encountered in lethally copper-stressed *E. coli* cells [29].

Compared to that of other transition metal ions, the higher affinity of copper ions for protein binding sites can lead to the replacement and/or displacement of native metals in metalloproteins when copper ions are present in excess within the microbial cell. This process is sometimes called mismetallation of proteins/enzymes and leads to their inactivation. Key metabolic pathways can thus be disrupted and induce the bacterial cell death [12,37]. Also, Cu^+^ has been shown to be more toxic for bacterial cells than Cu^2+^ [19,33]. This higher toxicity has been linked to a greater cytoplasmic membrane permeability for Cu^+^ than for Cu^2+^, and to the upper thiophilicity of Cu^+^ leading to a better ability of Cu^+^ to displace and/or destroy iron-sulfur clusters [30,39,46,47]. Macomber and Imlay first showed in *E. coli* that the enzymes dihydroxyacid dehydratase and isopropylmalate isomerase (IPMI) were inactivated in the presence of copper ions [30]. Furthermore, purified fumarase A enzyme was inactivated by Cu^+^ (and to a lesser extent by Cu^2+^) ions independently of ROS generation. This inactivation in the presence of copper ions was linked to the loss of iron atoms from the enzyme active site or to the downright destruction of iron-sulfur clusters within the enzymes. Another study later showed in *E. coli* that copper efflux could be induced to protect iron-sulfur clusters [47]. In Gram-positive bacteria, an excess of copper in *Bacillus subtilis* was shown to upregulate the expression of genes involved in iron-sulfur cluster scaffolding proteins (including *sufU*) as well as target enzymes of iron-sulfur cofactors. SufU protein is essential to iron-sulfur biogenesis and its upregulation is therefore a sign of a compromised iron-sulfur cluster assembly under copper-rich conditions [46]. It was furthermore demonstrated that Cu^+^ was able to destroy iron-sulfur clusters in recombinant SufU [46]. However, if the displacement of iron-sulfur clusters from key metabolic enzymes is thought to be the dominant mechanism for the bactericidal activity of copper ions in solution during chronic copper stress, the lethal pathway in contact killing with copper arising from solid surfaces (also termed acute copper stress) is thought to be different [19].

Copper-induced protein damages can also occur outside of the enzymatic active site. Structure disruption through copper-mediated formation of thioether and disulphide bonds can, for example, take place and lead to inactivation of the enzyme. Alternatively, copper-induced misfolding of proteins has also been reported [37].

### 2.4. DNA Damages

The occurrence of DNA damages has often been discussed as a primary or secondary event in copper-induced contact killing. Several early studies underscored DNA degradation as a main contributor to the killing of Meticillin-Susceptible and -Resistant *Staphylococcus aureus* (MSSA and MRSA) and Vancomycin Resistant enterococci (VRE) [28,32,43]. In *E. coli* O157:H7 and *Salmonella* spp., DNA damages appeared as delayed compared to observations made on Gram-positive bacteria [27]. Other studies even showed that no copper-mediated oxidation of DNA could be evidenced in *E. coli* or *Bulkholderia glumae* [21,25,40,42]. These discrepancies between Gram-positive and Gram-negative bacteria could be linked to the differences in their cell wall/membrane structures. DNA damages induced by exposition to copper ions are thought to occur mainly through the oxidative action of H_2_O_2_, O_2_^•−^ and OH^•^ [48]. However, in Gram-negative bacteria, the copper-catalyzed formation of OH^•^ takes place in the periplasm. Considering that the hydroxyl radical has an extremely short half-life (around 10^−9^ s) and that it is highly reactive, only molecules in close proximity to its production site would be likely targets for its oxidative action [49]. Therefore, DNA might be a too remote target for periplasm-generated OH^•^ in Gram-negative bacteria and/or be an indirect target needing a relay by other radicals to be attained. Another study indeed demonstrated DNA damages in a mutant *E. coli* strain with increased proportions of unsaturated fatty acids in the membrane [23]. Genomic DNA was absent in samples retrieved after an exposure to 99.9% copper of at least 45 min. On brass alloys containing 60 to 80% copper, DNA degradation was delayed and no genomic DNA destruction was evidenced after a 60 min exposure for brass with a 60% copper content. Moreover, DNA loss was found to occur after the bacterial cell death for brass alloys [23]. Similarly, comet assays showed that DNA fragmentation took place after cell death in an *E. coli* model [25].

## 3. In Vitro Antibacterial Activity of Copper and Brass Alloys

### 3.1. Vegetative Forms

**Figure 2:**
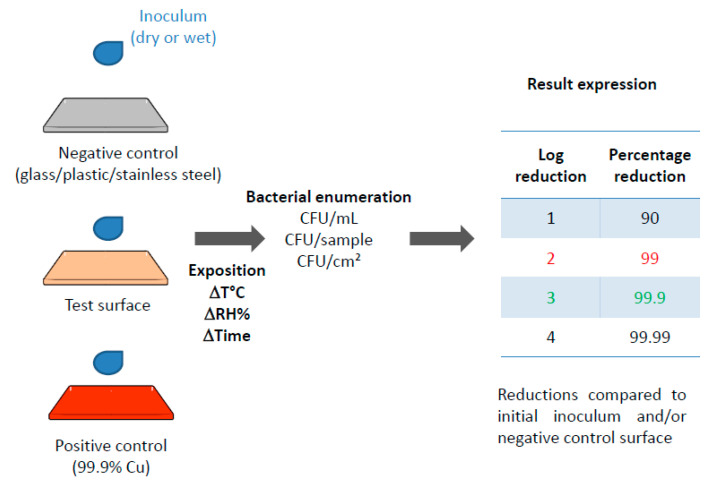
Antibacterial efficacy of copper-containing surfaces: general procedure and calculations. Reduction cutoff values compared to stainless steel for the validation of an anti-microbial efficacy according to Association Française de NORmalisation (AFNOR) NFS90-700 (in red) and US Environmental Protection Agency (EPA) (in green).

In order to demonstrate the antimicrobial efficacy of copper and copper alloy surfaces, a multitude of techniques have been used such as bacterial enumeration by culture, live/dead staining, fluorescence in situ hybridization or bioluminescent strains [50]. Despite varying experimental protocols, most studies relate a good efficacy of copper surfaces on vegetative forms of a wide range of bacterial species. The antibacterial efficacy is usually assessed after a time of exposure to the copper-containing surface using reduction in bacterial counts either compared to the initial inoculum and/or to the counts obtained on a control surface deprived of antibacterial properties such as glass, plastic or stainless steel (Figure 2).

These tests were first held on food pathogens and collection strains susceptible to antibiotics [51,52,53], but publications soon moved on to report significant reductions in bacterial counts on copper and copper alloy surfaces for a range of multidrug-resistant (MDR) bacteria and/or bacteria originating from clinical settings [32,54,55,56,57,58,59,60,61,62,63,64,65,66,67,68,69]. Indeed, the importance of testing clinical strains to evaluate whether any co-selected, cross-selected and/or co-regulated resistance between copper and antibiotics and/or detergent-disinfectants could occur soon appeared as mandatory [62]. Most of these studies gave encouraging results as to the efficiency of brasses on VRE, MDR or Extremely drug-resistant (XDR) *Acinetobacter baumannii*, Meticillin-Resistant *Staphylococcus aureus* (MRSA), MDR *Pseudomonas aeruginosa*, *Escherichia coli*, *Mycobacterium tuberculosis* and Carbapenem-producing enterobacteria (CPE) (Table 1). However, within a given bacterial genus, some species (and even within a given species, some strains) could display slightly different susceptibility to copper and brass alloys.

**Table 1:** Antibacterial activity of brass surfaces on selected nosocomial pathogens.

Bacterial Species	Copper Alloy (% Cu)	Efficacy ^a^		References
		Exposure Time		
		Short (≤1 h)	Long (>1 h)	
*S. aureus*	C26,000 (70)	98.8–100	ND–100	[67,68]
MRSA	C24,000 (80) C26,000 (70) NP (62–63)	74–100 6.7–97.3 6.7–99.85	84.1–100 ND–100 ND–100	[54,55,57,58,61,64,66]
*E. faecalis*	C26,000 (70) C28,000 (60)	~20 to 50 ~20	100 100	[28]
*E. faecium*	C26,000 (70) C28,000 (60)	~20 to 84 ~20	99.99–100 99.94–99.99	[28]
VRE	NP (62.5)	99.92	ND	[61]
*C. difficile* (vegetative cells and spores)	C26,000 (70)	36.9	68.4	[63]
*A. baumannii*	C26,000 (70)	0–98.3	98.1–100	[60,68]
MDR/XDR *A. baumannii*	C27,400 (63.2) NP (62–63)	0–99.85 20–99.95	0–99.94 ND–100	[55,57,58,60,61]
XDR *A. lwoffii*	C27,400 (63.2)	91.63	58.36	[60]
*A. pittii*	C27,400 (63.2)	68.89	99.79	[60]
MDR *Enterobacter* spp.	NP (62.5–63)	26–99.34	ND–100	[57,58,61]
*E. coli*	C21,000 (95) C23,000 (85) C26,000 (70) C28,000 (60) C83,300 (93) C83,600 (85) C85,700 (61)	>90 100 96.3–100 65.5–99.99 33.3–86.7 33.3 33.3	100 100 97.2–100 76.5–100 100 99.97–100 99.97–99.99	[27,52,53,66]
MDR *E. coli*	NP (62.5-63)	0–99.44	ND–98.4	[57,58,61]
*K. pneumoniae*	C26,000 (70) C28,000 (60) NP (62)	100 99.86–100 100	100 100 100	[27,55]
MDR *K. pneumoniae*	NP (62.5–63)	39.3–99.77	ND–73.2	[57,58,61]
*P. aeruginosa*	C26,000 (70) NP (62)	15.6–77.5 5.4	96.9–100 100	[55]
MDR *P. aeruginosa*	NP (62.5–63)	ND-100	97.2–100	[57,58,61]

Note: MRSA, Meticillin-Resistant *Staphylococcus aureus*; MDR, Multi-Drug-Resistant; ND, Not Determined; NP, Not Provided; VRE, Vancomycin-Resistant *Enterococcus*, XDR, extremely drug-Resistant; ^a^: % reduction against stainless steel (S30400) control (stated or calculated from data given in the references). Short exposure times ranged from 5 min to 60 min and long exposure times from 75 min to 24 h.

For example, within the *Acinetobacter* genus, the reduction in *Acinetobacter lwoffii* counts on brass obtained after a 5 h exposure were lower (around 99% reduction) than those obtained for *Acinetobacter pittii* and most *A. baumannii* strains (99.9% reduction and above) [60]. At the species level, one XDR *A. baumannii* strain count was unchanged after a 5 h exposure to brass, while the two other *A. baumannii* strains (1 MDR and 1 XDR) registered a count reduction of at least 99.9% [60]. Similarly, out of three MRSA strains, two were readily eradicated from the surface of brass while only a one log (90%) reduction was registered for the third one after a 6 h exposure [54].

A likely explanation for those behavioral discrepancies between strains of a single species is the development of adaptation mechanisms to cope with copper toxicity. Recent reports have shown that laboratory mutants of *E. coli* and *S. aureus* are able to survive on copper surfaces much longer than their parent strains [70]. However, the precise mechanism(s) leading to this increased survival could not be determined by proteomic and genomic analyses [70]. Upregulation of copper efflux systems such as Cus system in *E. coli* or transmission of plasmids harboring copper-resistance genes by Horizontal Gene Transfer (HGT) have been proposed to explain copper-tolerance in some strains [71]. Also, in *A. baumannii*, the presence of copRS in strains was recently linked with copper-tolerance [72]. All these mechanisms increase the survival time of strains on copper surfaces but do not prevent contact killing from eventually taking place. From an ecological point of view, no causal relationship between exposure to copper-containing surfaces and the de novo development of antibiotic-resistant strains has been evidenced so far. The likelihood of a widespread emergence of bacterial strains resistant to copper-induced contact killing and its possible contribution to overall antibiotic-resistance can be evaluated as low on the basis of the following considerations: (i) copper and copper alloys have been used for a very long time without enabling the rise of complete resistance to copper-induced contact killing, (ii) contact killing is a rapid process, especially in dry conditions, and does not leave bacterial cells the time to divide, and (iii) DNA damages, which are part of the contact-killing process, also target plasmidic DNA [16]. However, a point of concern is the possible contribution of these surfaces in the selection of strains already resistant to antibiotics and also carrying copper-tolerance/resistance genes through co-selection [71,73,74].

Other concerns regarding the use of copper-containing surfaces in general and brasses in particular as a mean to reduce the microbial bioburden in healthcare settings are the long-term persistence of a suitable antimicrobial effect, especially after repeated applications of disinfectants on these surfaces, and the effectiveness of disinfective treatments. A recent report indicated that antimicrobial efficacy of a solid copper alloy was better retained than those of a copper-containing polymer or a copper alloy-coated stainless steel [75]. A lower effectiveness of peracetic acid aerosol disinfection targeting *Geobacillus stearothermophilus* spores on copper and brass as opposed to ceramics, stainless steel or polyvinylchloride was also reported [76]. Thus, the impact of cleaning protocols and disinfectants on the antibacterial effectiveness of copper alloys warrants further work.

### 3.2. Sporulated Forms

On spore-forming bacteria, the results obtained by copper and brass surfaces were less clear-cut. For example, Weaver et al. [63] showed that copper and copper alloys containing at least 70% of copper were able to significantly reduce the number of *Clostridium difficile* vegetative forms and spores compared to stainless steel (Table 1). Similarly, no vegetative forms and less than 0.2% of germinating spores of two *C. difficile* strains were recovered from copper coupons after a 60 min exposure. However, no reduction in *C. difficile* dormant spores was recorded after a 3 h exposure [69]. Aerobic spore-forming *Bacillus subtilis* was also employed as a test species to explore the sporicidal activity of copper and of a silver nickel copper alloy (solid or sprayed forms). It showed a significant reduction in dormant and germinating spores [77]. These results were not in accordance with an earlier report that only registered a 1 log reduction in *B. subtilis* counts after a 45 min exposure. By contrast, a *B. subtilis* mutant strain unable to form endospores was eradicated from copper and brass after a 20–30 min exposure, indicating that endospores were instrumental in the survival of *B. subtilis* on the surface [24]. *Bacillus anthracis* vegetative forms were found to be slightly reduced (−1 log) on copper as compared to stainless steel after a 10 min exposure but *B. anthracis* counts remained steady afterwards during the full 24 h exposure time [41]. This persistence was once more demonstrated to be linked with the presence of *B. anthracis* endospores [41]. Espirito-Santo et al. also showed that endospores from *Bacillus cereus* were able to survive after a short exposition to copper [25]. Taken together, those results imply that mechanisms involved in contact killing are less efficient on endospores than on vegetative forms.

### 3.3. Factors Influencing the Antibacterial Effectiveness of Copper Alloys

The experiments reported above use varying sets of parameters (inoculum density and volume, wet vs. dry inoculation, incubation temperature and relative humidity (RH), and so on). It is therefore difficult to draw fair comparisons and give a ruling on the relative efficacy of different copper alloys from published results, if these alloys are not tested within the same framework. While 99.9% “pure” copper is widely confirmed and used as the “gold standard” of antibacterial efficacy, the ranking of antibacterial activity according to the copper content of alloys is not as straightforward. Looking at the results of a number of laboratory studies published so far, the greater the copper content of a copper surface, the better and/or quicker the reported antimicrobial efficacy appears to be [23,52,59,66,78,79]. However, MacDonald et al. showed that bronze containing 95% copper was less efficient than 70% copper brass and 99.9% copper in reducing *S. aureus* viable counts [68]. Similarly, Noyce et al. pointed out that aluminum-bronze C95,500 (78% Cu) and nickel-aluminum-bronze C9,5800 (9% Al, 81% Cu) demonstrated poor antimicrobial effectiveness in spite of their relatively high copper contents [54]. Also, similar antibacterial activities against *C. albicans* and *K. pneumoniae* were reported for brass alloys containing 62.5 and 70% copper [55] while *Cronobacter sakazakii* was as readily eradicated on brass and copper nickel containing 70% and 88.6% copper as on 99.9% copper under dry conditions [80]. The antibacterial activity against *Salmonella* strains was also reported as being greater on brass containing 60% copper than on nickel silver containing 65% copper [78]. Therefore, copper content might not be the only parameter driving the antibacterial effectiveness of copper alloys. Pointing out the occasional better antibacterial properties of nickel-silver C75,200 (nickel-silver) against cartridge brass C26,000 with a higher copper content, Warnes et al. suggested that other metal constituents and physical properties (especially copper-release rates) may have a role in the bactericidal activity of copper alloys [28].

Other parameters have also been shown to influence the antibacterial effectiveness of copper-containing surfaces such as:

- Surface structure

Cold spray deposition of copper led to a higher reduction in MRSA than those witnessed for plasma and wire arc depositions [81]. Also, a copper surface generated by electroplating has been shown to generate a twofold higher copper release in the medium compared to rolled and polished coppers [82]. This higher rate of copper release was correlated with a swifter antibacterial activity of the electroplated copper [82]. The authors also hypothesized that the grooves generated through electroplating allowed for an enhanced contact surface with the bacteria that could, in turn, favor the contact-killing process.

- Surface oxidization

Under biospheric conditions, both CuO and Cu_2_O can be formed by oxidization on the surface of copper-containing materials [83]. Cu_2_O production is favored by a reducing environment (e.g., in the presence of organic matter or bacteria) while CuO is mainly generated in an oxidizing environment. Both cuprous and cupric oxides have been shown to display antibacterial activity but CuO was reported as less effective than Cu_2_O, which has an antibacterial activity similar to that of copper [84]. Also, treatments lowering the corrosion process resulted in a lower antibacterial effectiveness of copper surfaces [36].

- Temperature and RH

MRSA were shown to be as readily killed under high temperature and RH (35 °C and 90% RH) as under low temperature and RH (20 °C, 24% RH) [64]. However, pointing out that the 37 °C and 100% RH conditions recommended in the ISO 22196 [85] antimicrobial surface efficacy test were far from reflecting actual healthcare environmental conditions, Ojeil et al. proposed to test the antimicrobial activity of copper surfaces at either 20 °C, 50% RH or 20 °C, 40% RH [67]. They showed that the higher temperature and RH conditions allowed for a greater antimicrobial efficacy on a *S. aureus* strain of all copper alloys tested than those closer to actual environmental conditions. Noyce et al. also reported the differences in antibacterial activities against *E. coli* after exposures at 4 °C and 22 °C [53]. In this work, most copper alloys (including three brass alloys) allowed for a faster complete kill at 22 °C than at 4 °C.

- Wet or dry inoculum/exposure

One of the most influencing features in the way antibacterial testing is performed for copper-containing surfaces is the inoculum volume, whether it is spread or not and whether it is kept moist. One the one hand, dry inoculation of the bacteria (small volume, spread and dried) on the surface is generally thought to be more representative of real-life conditions [86]. It is also a better way to take into account the susceptibility of bacterial strains to desiccation on non-porous surfaces. On the other hand, wet exposure is more conducive to bacterial growth on surfaces than dry exposure and would represent a worst-case scenario.

Therefore, wet and dry conditions have been tested for the inoculation of bacteria on copper-containing surfaces and the results compared in several papers. Espirito Santo et al. were among the first to report that a reduction in inoculum moisture led to a decreased survival of *E. coli* on copper-containing surfaces as compared to previous studies using wet inoculums conditions. This reduction could not be linked to desiccation or osmotic stress as the strain displayed good survival rates on stainless steel when dry inoculum conditions were applied [21]. However, the same team showed that Gram-positive bacteria survived longer than Gram-negative ones under dry inoculum conditions [87]. This pattern was later confirmed on *E. faecium* [36]. Using *C. sakazakii* strains, another study showed that dry inoculation conditions (2 µL of the bacterial suspension spread on the surface and left to dry in open air) enabled a swifter reduction in viable bacterial counts than under wet inoculation conditions (25 µL of the bacterial suspension spread on the surface kept in a closed container to maintain moisture) [80]. Also, comparing a 9 µL spread inoculum with a 1 µL non-spread one, Dauvergne et al. showed that the bacterial recovery was greater with the later technique [61].

- Presence of organic compounds

As the main mechanism of action for copper-containing surfaces is contact killing, which is thought to be driven by the release of copper ions, the presence of organic molecules on the surface could interfere with the action cupric and cuprous ions on bacteria. Indeed, the addition of liquid beef extract reduced the antimicrobial effect of several copper alloys including brass against *E. coli* [53]. The authors of this study hypothesized that beef extract might act as a protective matrix against copper exposure, especially because of its fat content. *C. sakazakii* reduction rates were also found to be lower when bacteria were suspended in an infant formula as compared to Tryptic Soy Broth (TSB) [80]. Using repeated soiling with *S. aureus* suspended in 1% Bovine Serum Albumin (BSA), a study showed that despite cleaning procedures, the efficiency of a copper surface against *S. aureus* was reduced [88]. However, Ojeil et al. later showed that the addition of BSA at 3 g/L did not significantly modify the antibacterial activity of a series of copper alloys against *S. aureus* [67]. Some copper alloys even displayed a better efficacy when the BSA soil load was present [67]. Another work demonstrated that the presence of organic compounds such as those contained in TSB impaired the antibacterial activity of copper and brass against *E. coli* and *S. aureus* [89].

All these influencing parameters led to the publication of various standardized or normalized protocols to assess the antimicrobial activity of non-porous surfaces and allow fairer comparisons of efficacies between studies, such as:

- International Organization for Standardization (ISO) 22196:2011 [86]

As described above, this guideline recommends antibacterial activity testing in temperature and RH conditions that are far from healthcare settings’ ones. Also, a wet inoculum of 0.4 mL is used in this protocol, which is not in line with the usual volumes of droplets bearing microbial contaminations in the environment. Moreover, this protocol was originally designed to test plastic surfaces and has an exposure end point of 24 h. Such a distant end point might not be adapted to simulate real-life conditions for metal surfaces present in healthcare settings and more especially for copper and brass because of their rapid antibacterial activity.

- Environmental Protection Agency (EPA) protocols [90]

In 2008, the American EPA published a list of copper alloys validated as suitable for claiming antimicrobial properties on the basis of a series of tests. The first test to be passed in order to be coined as a sanitizer is a reduction of at least 99.9% (3 log) of a dry (20 µL) bacterial inoculum on said surface after a 2 h exposure (Figure 2). Two additional protocols have also been validated by the EPA: the residual self-sanitizing activity test (held after cycles of wet and dry wear) and the continuous reduction of bacterial contamination test (including 8 repeat inoculations on the metal surface over a 24 h period).

- Association Française de NORmalisation (AFNOR) NF S90–700 [91]

Recently, a French norm has been published on the bactericidal effectiveness of non-porous surfaces [91]. The method described therein mentions a dry inoculum of 1 µL, an exposure time of 1 h and a cutoff for the validation of antibacterial properties of a 99% (2 log) reduction (Figure 2). This cutoff value is less stringent than that of EPA but NF S90-700 only allows one hour before evaluating the residual viable bacteria whereas EPA has a 2 h exposure time. These two protocols therefore do not agree on the reduction to be attained and other authors have proposed [57] that a reduction ranging from 2 to 3 log (99 to 99.9%) would mean bacteriostatic properties for the antimicrobial surface while a reduction of over 3 log (>99.9%) would stand for bactericidal properties.

## 4. Field Demonstration of the Antimicrobial Efficacy of Copper and Brass Alloys

The first report of the antibacterial properties of brass in hospital settings was published by Kuhn in 1983 [14]. This paper highlighted that, even though the door hardware made of brass looked dirtier than the stainless steel one, it limited the bacterial bioburden and could be a mean of reducing HAIs. There is a gap of over 20 years between this first observational study and the publication of further field trials attempting to ascertain the efficacy of copper and brass alloys in reducing the bacterial bioburden on commonly touched surfaces. The possibility of transmission of nosocomial pathogens from environmental sources and more specifically from frequently touched surfaces had indeed gained credibility in the meantime [92,93]. To this day, trials on bioburden reduction outnumber the ones seeking to establish a link between this reduction and a possible decrease in the prevalence and/or incidence of HAIs, as will be seen below.

Most of the studies using copper alloys and presented below were implemented in intensive care units (ICUs) to limit the inherent biases linked with field trials. For example, critically ill patients are generally not ambulatory. Therefore, interactions of these patients with other environmental surfaces within and outside the room are limited. Additionally, ICU patients are at further risk of HAI because of the severity of illness, invasive procedures, and frequent interactions with healthcare workers [94].

### 4.1. Reduction of the Global Bacterial Bioburden on Hospital Surfaces

In 2010, a study held in a German hospital by Mikolay et al. reported a significant one-third reduction in the bacterial bioburden on brass doorknobs as compared to aluminum ones [13]. However, no changes were witnessed for the bacterial load harvested from brass push plates and light switches (only in the winter test period for the latter). The authors explained this lack of significant reduction by stating that the bacterial load recovered was 5 to 6 times lower on push plates and light switches as compared to doorknobs. After cleaning, a significant reduction in the recolonization speed of doorknobs was also witnessed. The authors also pointed out the need for an efficient cleaning procedure in order to promote and maintain the bioburden reduction seen on brass alloys [13]. The same year, Casey et al. [95] also investigated the impact of brass alloys in a cross-over study held in Birmingham (UK). The design of this study enabled to mitigate possible biases that could be pointed out in the previous one, e.g., a difference in the microbial contamination of the tested surfaces linked to their location and frequency of use. Indeed, after 5 weeks of investigation, locations of the control and brass alloy surfaces were switched, hence reducing the possible impact of such a bias. Also, copper-containing materials were implemented in the ward 6 months prior to the study start, allowing patients and staff to become accustomed to these new materials. The main outcome was a significant reduction in the median number of bacteria found on the three copper-containing tests surfaces as compared to their respective controls. Indicator pathogens were also investigated and retrieved only once from copper-containing surfaces. However, this was a small-scale pilot study and a possible causative link with the colonization/infection of patients housed in this ward was not investigated [95]. Working in a primary healthcare clinic, Marais et al. [96] also concluded on a significant reduction of the bacterial bioburden on copper-containing surfaces compared to their respective control. The frequency at which the surface was likely to be touched by patients and/or staff was not reported to influence the significance of the witnessed reduction. The only situation in which the bioburden was comparable between copper surfaces and their control counterparts was over weekend periods (71 h) when the clinic was closed and microbial loads markedly reduced [96]. In Chile, a copper-based alloy was used for bed rails, over-bed tables, chair arms, intravenous (IV) poles, and bed levers in a controlled study held in a medical ICU to try and reduce the *S. aureus* bioburden [97]. The authors witnessed a reduction in the bioburden of at least 82% on tested copper surfaces as compared to their controls. The ambient humidity conditions for this trial were quite dry as humidity levels typically range between 7 and 20% in the semi-desertic region in which the Chilean hospital was located.

These early reports on the effectiveness of brass alloys and other copper-containing materials displayed great variations in study protocols with possible biases and/or a lack of proper correlation between the reduction in bacterial bioburden and the incidence of HAIs. It soon led to the publication of a position paper advocating for standard requirements and a common methodology to evaluate new materials for controlling HAIs [94]. Around the same time, a microbiological standard for defining bacterial cleanliness of maximum 2.5 CFUs/cm² was also proposed [94,98]. Sharpe et al. [94] advocated for investigations to be held in various population groups (elderly, immune-compromised and general populations) and settings (e.g., single-occupancy vs. multiple-occupancy rooms, ICUs vs. general wards, elder care vs. hospital buildings). They also pointed out the importance of evaluating the risk reduction of acquiring a HAI in conjunction with the evaluation of the reduction in the surface bioburden [94].

The following year, the results of another small-scale study were published. In an outpatient clinic, the upper surface of the wooden arms of two phlebotomy chairs was fitted with brass along with the plastic trays attached to the chairs [99]. Compared to a control chair with unchanged arms and tray, a reduction in the overall bioburden of at least 88% was obtained. Although a rotation in the location of the three chairs was implemented every 5 weeks to account for unequal frequencies of use, several limits could be pointed out for this study: (i) the low number of surfaces tested, (ii) the study was not blinded, which could have induced a bias in the cleaning of the chairs, and (iii) patient characteristics that might influence the bacterial bioburden were not evaluated.

Most of the studies described above were pilot studies, with a small number of surfaces included [95,96,99]. In 2012, the results of a much larger study including 6 objects in 16 rooms of ICUs in three different hospitals over 43 months were published [100]. A strength of this study was the beginning of sampling 23 months prior to the implementation of copper-containing surfaces, hence reducing a possible bias induced by a change in cleaning practices when sampling is known to take place. Indeed, a surprising 64% decrease in the microbial burden between the pre-intervention and intervention phases in the control rooms was found. Several independent and uncontrolled variables were put forward by the authors to explain this result such as a better cleaning of the environment by the staff in units in which copper materials were present, a so-called antimicrobial halo effect of copper that limited the transfer of microbes between control rooms (as staff were common to both rooms), and/or variations in compliance with other infection control measures such as hand hygiene. It also has to be highlighted that, even though a significant reduction in the global bacterial bioburden was witnessed on these high-touch items as compared to the controls, brass was not the only copper alloy used in these experiments. A companion study of this work on the prevention of HAIs and colonization of these ICUs patients was released the following year and will be described below [101].

Karpanen et al. [102] published the results of a cross-over study held in an ICU. Copper-containing materials were switched with their control counterparts at 12 weeks after a 4 month “washout” period to reduce the bias of usage patterns. Of the fourteen copper items tested, only 5 were made of brass alloys. In addition to estimating the bacterial bioburden on copper-containing touch surfaces, it is the first study reporting the number of patients infected or colonized by nosocomial pathogens, as will be detailed in Section 4.3. The study design also took into account several potential interfering factors such as the staffing of the ICU, the hand-hygiene compliance and the mean bed occupancy. No significant differences were observed between the two 12 week periods. All but one (grab rails) of the 5 items made of brass alloys displayed a significant reduction in their bacterial bioburden as compared to their control counterparts. The lack of significance registered for grab rails could be explained by low basal levels of bacterial contamination and it was therefore difficult to achieve a significant reduction with the brass alloy.

Schmidt et al. [103] worked on the bioburden of the sole bedrails, arguing that these devices were at the cross-road of potential contaminations by patients, healthcare workers and visitors. This study was held on a limited number of beds but took into account the impact of cleaning practices by sampling the devices once before and multiple times after the daily cleaning routine. A significant reduction in the bacterial bioburden was witnessed on copper bedrails at each sampling time except for the one at 30 min post-cleaning. Once more, this lack of significance could be attributed to the impact of cleaning. The cleaning procedure induced lower counts in bacteria on plastic detected at this time point. The authors also pointed out a lower frequency of bacterial contamination above the threshold of 2.5 CFUs/cm² on the copper surface. The same team afterwards worked on active care beds with surfaces close to the patient encapsulated in copper [104]. Their findings included a bacterial contamination of these surfaces correlated with the patients’ length of stay, a significant reduction in the bacterial contamination on copper-bed surfaces, and once more a lower frequency of bacterial bioburden above the 2.5 CFUs/cm² threshold for the same beds [104].

Ruelle et al. [105] focused their work on door handles implemented in a neonatal ICU and 2 pediatric units and showed a global reduction in the bacterial burden for brass door handles.

Schmidt et al. [106] extended their work on pediatric ICUs with the following rationale: patients from pediatric and neonatal ICUs are at higher risk of HAIs than typical adult ICU patients and multiple housing in often-used such pediatric and neonatal ICUs as opposed to single housing in adult ICUs. Along with the expected reduction in the bacterial bioburden on bedrails and faucet handles, this paper gives interesting evidence on the differences in bioburdens between occupied and unoccupied beds as well as on the reduction in post-intervention bioburden in control rooms as compared to the one obtained pre-intervention. This latter point once more advocates for the so-called “halo” effect of copper-containing devices with a global reduction in bacterial contamination of surfaces. Results on the frequency of contaminations above a 5 CFU/cm² threshold were also found to be significantly lower on brass devices as compared to the controls. Above 5 CFU/cm², the risk of HAI has indeed been determined as significantly higher [101].

Some studies have also been carried out on healthcare settings outside of the hospital, such as long-term care facilities for the elderly [107,108,109]. A first study performed in Finland in various facilities (kindergarten, retirement home, pediatric unit in a hospital and an office building) reported consistent lower total bacterial loads on copper touch surfaces compared with their chromed, plastic or wooden reference surfaces. However, only two types of devices were made of brass (front door pullers in the office building and door handles in the kindergarten and retirement home) and the others of pure copper [107]. No significant reductions were found for the total bacterial burden on these brass surfaces. Door handles and corridor hand-rails made of copper alloy, the precise composition of which was not described, were also implemented in 5 long-term care facilities in France and their bacterial bioburden investigated after a minimal time of use of 22 months [108]. A significant reduction in the bacterial bioburden was typically found on copper door handles and handrails as compared to their control counterparts. This paper also highlighted variations in the bioburden between healthcare facilities. Moreover, the authors reported that door handles made of copper alloy used for three years in these facilities, although still inducing a significant reduction in the survival of a MRSA strain compared to stainless steel, were less efficient than similar unused door handles, highlighting a potential impact of wear on the antimicrobial activity [108]. In a subsequent paper, the same team identified the main contributors to the bacterial burden on their samples as belonging to the *Staphylococcus* and *Micrococcus* genera [109].

### 4.2. Reduction in Global Bacterial Bioburden on Ancillary Surfaces

The microbial contamination of 25 pens made of brass (CuZn15) and 25 stainless steel ones used by nurses in a British ICU has been compared [110]. After a 12.5 h shift, “single use” brass or stainless steel pens were sent to the laboratory and immediately sampled to evaluate the microbial bioburden. Similarly, another set of 50 “single use” pens was collected and stored at room temperature for 11 h to simulate nonuse between shifts before pens were sampled. Sampling just after use showed that the number of contaminated pens did not significantly differ between brass and stainless steel (48% vs. 68%, respectively). However, a significant reduction in the median number of recovered CFUs per pen was found, be it immediately post-use or after the 11 h storage. Additionally, brass pens stored for 11 h were less frequently contaminated than stainless steel ones (20% vs. 72%, respectively). Bacteria recovered from pens were mostly coagulase-negative staphylococci and micrococci, whatever the metal they were made of [110].

Stethoscopes made of copper alloys were also investigated in a prospective study on 21 healthcare providers (14 in a pediatric emergency unit and 7 in adult medical-surgical settings) blinded as to the purpose of the study [111]. Healthcare providers used the control and the copper-containing stethoscope for one week prior to sampling for the bacterial bioburden evaluation. The total bacterial aerobic counts recovered from three sampling sites of stethoscopes made of copper alloys (including C87610 copper-silicon alloy for the chest piece) was significantly lower than that of their copper-free homologues (including stainless steel for the chest piece) independent of the healthcare provider or infection control practices. A significant reduction in the frequency of mannitol-positive staphylococci was also reported [111].

### 4.3. Reduction in Specific Bacterial Groups

Some studies have attempted to identify the bacteria found on copper and brass surfaces [99,100,102,105,108,109]. Staphylococci were reported as the most frequently recovered bacteria on these surfaces [99,100], along with *Streptococcus* spp., *Roseomonas* spp. [109]. Studies have then tried to find a significant difference in contamination frequencies of several nosocomial pathogens such as MRSA, VRE or coliforms between control surfaces and copper-containing ones [99,100,102,105,109]. However, such attempts were not always successful because the isolation frequencies on control surfaces were sometimes low for these pathogens, especially for VRE and coliforms [109]. Nevertheless, Karpanen et al. managed to describe a significant reduction in VRE, Meticillin-Susceptible *S. aureus* and coliforms on various copper alloys, including brass [102]. Similarly, Ruelle et al. showed a reduction in colonization frequencies for *S. aureus* on brass door handles [105], while Colin et al. witnessed significant reductions in the frequencies of isolation for *Staphylococcus* spp., *Streptococcus* spp., *Roseomonas* spp. on copper alloy surfaces as compared to control ones [109]. However, the antibacterial activity of the same copper surfaces against MRSA was lowered after three years of regular use but still significant compared to controls [108].

### 4.4. Brass Alloys to Reduce Hospital Acquired Infections?

The first proper attempt at evaluating a possible reduction in the incidence of HAIs using copper-containing alloys was published by Salgado et al. [101]. The authors registered a significant reduction in HAIs and/or colonization by MRSA and VRE in ICUs (Table 2) in a prospective, intention-to-treat study in room containing 6 objects made of copper-based alloys vs. rooms with control objects. There was no influence of the length of stay on the occurrence of HAI/colonization in either type of rooms. A significant relation between environmental burden and the occurrence of HAI was also witnessed [101]. Limitations were nevertheless highlighted such as the removal of some copper-containing objects from patient rooms or the introduction of such objects in control rooms. Also, the study was not double-blinded, but such a study design would be difficult to achieve because of the distinctive aspects of copper alloys and their control counterparts. Modifications in hand-hygiene routines following the introduction of copper-containing objects have also been pointed out as a possible interfering factor. However, in this case, a 9 month gap existed between the first implementation of these copper-containing objects and the start of the study, hence mitigating the influence of this putative interfering factor. Questions about the possibility of selective reporting and the biological plausibility of the findings were also raised [112].

Another prospective study on the possible reduction of HAIs incidence was held in Chile [113] in an adult intensive care unit (Table 2). Despite a lower rate of catheter-associated bacteremia in copper-fitted rooms, no significant difference in HAI global incidence was found between rooms fitted with copper and control rooms. However, several limitations have been put forward by the authors themselves such as the limited number of included patients compared to the one forecast to get a significant statistical result or the fact that infections not related with invasive devices were not taken into account. Others can be added such as a minimum length of stay to be included in the study of 24 h, when HAIs are usually diagnosed after a 48 h stay. Also, different cleaning protocols were used for copper-based devices (0.6% citric acid) and their counterparts (quaternary ammoniums).

In a companion study to the one held in pediatric intensive care units on bacterial bioburden by Schmidt et al. [106], the authors failed to find a significant reduction in HAI rates after the implementation of brass devices (Table 2) [114]. Nevertheless, HAI incidence rates decreased from 13.0 per 1000 patient days for patients treated in the control settings to 10.6 per 1000 patient days for patients treated in intervened rooms. Patients who developed HAI events in copper-containing rooms more frequently presented pre-existing conditions and had longer lengths of stay than the ones in control rooms [114]. Despite the occurrence of several interfering factors such as temporary overcrowding of the pediatric ICU and assumptions for the statistical analysis that were unmet, these results of this trial are still interesting. The authors point out the methodological difficulties encountered in this kind of environmental interventions and also the potential economical interest in implementing antimicrobial copper surfaces in addition to the public health one [114].

**Table 2:** Main characteristics of field trials on the reduction of hospital-acquired infections by brass alloys and copper-containing materials.

Setting (Country)/Study Design	Location (Alloy/Cu material vs. Control Material)	Patient Numbers	Study Length	Evaluation Criteria	Main Outcomes	Reference
Intensive Care Units in 3 hospitals (United States)/ Prospective, intention-to-treat	4 common (not specified vs. not specified): - Bed rails - Overbed tables - IV poles - Arms of visitor chairs 2 variables (depending on the hospital)	320 controls vs. 294 tests	July 2010–June 2011	HAI and/or colonization by MRSA/VRE frequencies	HAIs + MRSA/VRE colonization significantly reduced (*p* = 0.02) HAIs alone significantly reduced (*p* = 0.013)	[101]
1 adult Intensive Care Unit (Chile)/ Prospective	3 (99% Cu coating vs. not specified): - Bed rails - Overbed tables - IV poles	217 controls vs. 223 tests	May 2011–May 2012	HAI frequency	No significant difference	[113]
2 pediatric intensive care units (Chile)/Prospective, intention-to-treat, non-randomized, controlled	- Bed rails/bed rail levers (62% Cu brass/85% Cu brass vs. polypropylene/not specified) - IV poles (Not specified vs. not specified) - Faucet handles (73% Cu brass vs. stainless steel) - Surface of healthcare workstation (62% Cu brass/85% Cu brass vs. not specified)	254 controls vs. 261 tests	November 2012–November 2013	HAI incidence rates	No significant difference	[114]
1 long-term care facility (France)/Prospective, longitudinal, observational pilot study	Door handles (90% Cu copper alloy vs. polyvinylchloride) Handrails & grab bars (70% Cu copper alloy vs. wood)	289 controls vs. 267 tests	February 2015–June, 2016	Relative risk of HAIs during outbreaks	No significant difference	[115]

Lastly, a study held in one of the long-term care facilities fitted with copper alloy door handles and handrails presented in the studies by Colin et al. [108,109] showed no significant differences in the relative risk of infections during nosocomial outbreaks between the copper-fitted wing and the control wing [115] (Table 2). However, when results were split according to the transmission mode of the causative agents (suspected to mostly be viruses), a significant reduction of the relative risk of infection was witnessed in the copper outfitted wing for hand-transmitted infectious agents (*p* < 0.001), which appears as logical.

Most of the abovementioned studies are objectionable, mainly because randomized double-blinded studies are impossible to achieve when replacing a surface by another one displaying distinguishable features. However, to strengthen the evidence for the use of antibacterial brass surfaces in healthcare settings, further field works taking into account the TREND and ORION statements should be carried out [116].

## 5. Conclusions

The mechanisms underlying the antimicrobial effects of copper-containing surfaces are now fairly well described, even if the prevalence and order in which these mechanisms take place are still a matter of debate. Many laboratory studies have been held on copper-containing surfaces. They show a broad spectrum of activity for these surfaces against bacteria. Only against bacterial spores is their effectiveness limited. However, these laboratory trials point out that various parameters related to the surface structure, environmental conditions, inoculums and presence of organic soils on the surface can mitigate the antibacterial effectiveness of brass and copper surfaces.

Field trials using brass and copper surfaces consistently report reductions in the bacterial bioburden, but evidence is still sparse as to a significant impact on HAIs. To better establish the impact of those surfaces on HAIs, further studies are warranted. Similarly, further work is still needed to assess the long-term effects of chemical/physical wear on the antimicrobial effectiveness copper and brass surfaces. Indeed, not all copper-containing surfaces are equal. Depending on the copper alloy composition, soiling and tarnishing might occur at varying speeds, sometimes causing acceptance problems [117]. Also, the advantages of integral brass alloys against CuO containing resins should be justified by additional studies. The latter ones have recently been developed to benefit from the antimicrobial activity of copper ions with a less-costly material. However, some experiments already showed that their effectiveness decreases with time and wear as compared to integral copper alloys [75,118]. Brass touch-surfaces should therefore be considered a complement to, not a substitute for, hand-hygiene practices, disinfection operations and other standard cleaning methods.
